# Non-ST Segment Elevation Myocardial Infarction Associated With Cocaine Use

**DOI:** 10.7759/cureus.32875

**Published:** 2022-12-23

**Authors:** Ishan Choksey, Thor S Stead, Rohan K Mangal, Carlos Lopez Ortiz, Latha Ganti

**Affiliations:** 1 Biology, Trinity Preparatory School, Winter Park, USA; 2 Medicine, Warren Alpert Medical School of Brown University, Providence, USA; 3 Medicine, University of Miami Miller School of Medicine, Miami, USA; 4 Emergency Medicine, University of Puerto Rico, San Juan, PRI; 5 Emergency Medicine, HCA Florida Ocala Hospital, Ocala, USA; 6 Emergency Medicine, Envision Physician Services, Plantation, USA; 7 Emergency Medicine, University of Central Florida College of Medicine, Orlando, USA

**Keywords:** acute myocardial infarction, chest pain in the young, cardiac troponin, cocaine-induced coronary disease, non-st segment elevation myocardial infarction (nstemi)

## Abstract

We present the case of a 23-year-old male with no significant past medical history who experienced acute chest pain. A diagnostic workup revealed that the patient had a non-ST-segment elevation myocardial infarction. Although the patient was not forthcoming initially with his cocaine use, he did admit it later in his emergency department course. The timing of his rise and fall of troponin is presented. The pathophysiology of cocaine-related chest pain and infarction is discussed. The patient continued to make an uneventful recovery.

## Introduction

Although acute myocardial infarction (AMI) in adults under the age of 30 years is not common, it is becoming more prevalent [1]. A prospective case series of 1,116 patients in India aged less than 30 years revealed that smoking (78.5%), family history of premature coronary artery disease (CAD) (46.8%), obesity (39.1%), physical inactivity (38.7%), and stressful life events (29.6%) are the most common risk factors for AMI [2]. Non-ST-segment elevated myocardial infarction (NSTEMI) is the most common presentation across the spectrum of AMIs and is also brought on by many of the same risk factors, including smoking and cocaine use [3].

Cocaine is a very powerful stimulant drug that is obtained from the *Erythroxylon coca* plant. It has been used in South America as a mild stimulant akin to coffee for thousands of years in the form of chewed coca leaves [4]. Cocaine hydrochloride is a purified chemical that was isolated in the early 1900s and used as an analgesic prior to the development of anesthetics as a class [5]. However, cocaine is notable for its cardiotoxic effects such as suppressing myocardial contractility as well as reducing coronary caliber and coronary blood flow [6]. Furthermore, a review conducted in 2005 found that 57% of cocaine-related emergency department (ED) visits due to chest pain result in hospital admission [7].

According to the 2020 National Survey on Drug Use and Health [8], approximately four million Americans aged 12-30 years used cocaine in the past year. In 2021, cocaine-related chest pain visits to the ED were highest among patients aged 26-44 years (45.5%), followed by patients aged 45-64 years (38.6%). Males accounted for 71.7% of cocaine-related ED visits. With regard to ethnicity, Blacks comprised the highest percentage at 44.2%, while Whites accounted for the second highest percentage of these visits (35.1%). The majority (80.7%) were non-Hispanic. The regional percentage of cocaine-related ED visits was highest among the patients residing in the Northeast (26.8%), followed by patients residing in the West (13.6%) [9].

Differential etiologies of myocardial infarction in young adults should also include infectious causes, such as myocarditis, endocarditis, and pericarditis. ED presentation of endocarditis typically includes fever, tachycardia, and joint or muscle aches secondary to septic arthritis [10], whereas the ED presentation of myocarditis includes chest pain, arrhythmias including tachycardia, and shortness of breath [11]. Notably, COVID-19 has proven to be an important etiology of myocarditis in young adults, with a multicenter retrospective cohort study revealing 1.37 events per 100,000 individuals among 16-24-year-old males who developed myocarditis within 28 days of testing positive for COVID-19 [12]. Lastly, ED presentation of pericarditis typically includes coughing, fatigue, peripheral edema, and fever [13].

## Case presentation

A 23-year-old male presented to the ED due to chest pain that began earlier that morning. He also reported a mild headache, and he was doing nothing but laying down when it happened. He called in sick to work and came to the ED. The pain was located on the left side of the sternum, with point tenderness over the area. There was no history of trauma in that area, and there were no external bruising or open wounds.

The patient's only medical history was mild asthma. He had no family history of any cardiac disease. He had never had any surgeries. He did not take any medications on a daily basis.

His typical day consisted of waking up, brushing his teeth, taking a shower, and then going to work. He stated that he worked in finance and thus has a desk job. He admitted to getting very little physical activity. His body mass index was 25.9. He stated that he usually gets winded when climbing stairs and that this was nothing new for him.

The patient expressed a concern that his headache could be related to the dental work he had three days prior to his ED visit. He explained that he had a gingival infection, so his dentist had to do a deep cleaning and then a crown restoration. He did not receive pre- or post-prophylactic antibiotics for the dental procedure.

The patient’s initial vital signs included a blood pressure of 138/93 mmHg, oxygen saturation of 100% on room air, a temperature of 98.2 degrees Fahrenheit, a pulse of 61 beats per minute, and a respiratory rate of 16.

Physical examination revealed a well-appearing, well-developed male. He was awake, alert, cooperative, and had a non-toxic appearance, and did not have any acute respiratory distress. The pulmonary exam did not reveal any rales, rhonchi, wheezing, retractions, stridor, chest wall deformity, or crepitus. There was a point of tenderness at the central left side of the sternum. He had normal heart sounds, peripheral circulation, and a regular rate and rhythm. He did not have any gallop, murmurs, rubs, or gross differential in blood pressure when measured in each arm. His capillary refill was not delayed, and his pulses were equal bilaterally.

Given the history of recent dental work, the patient's relatively young age, and his lack of cardiovascular risk factors, endocarditis was high on the list of differential diagnoses, and an urgent echocardiogram was ordered in addition to the standard workup to rule out acute myocardial ischemia.

Laboratory tests (Table 1) revealed elevated glucose (113 mg/dL), aspartate aminotransferase (64 units/L), troponin I (4.31 ng/mL), C-reactive protein (1.84 mg/dL), and monocytes (0.8 k/mm^3^). Everything else was within the normal range.

**Table 1:** Patient's laboratory results NAA: Nucleic acid amplification.

Test	Results	Reference range
Sodium (mmol/L)	140	135–145
Potassium (mmol/L)	4.0	3.5–5.3
Chloride	105	98–107
Carbon dioxide (mmol/L)	29	21–32
Blood urea nitrogen (BUN) (mg/dL)	15	7–18
Creatinine (mg/dL)	1.2	0.6–1.3
Estimated glomerular filtration rate	>60	>60
Glucose (mg/dL)	113	70–110
Calcium (mg/dL)	8.8	8.4–10.2
Troponin I (ng/mL)	4.31	0.02–0.05
Total bilirubin (mg/dL)	0.7	0.0–1.0
Aspartate aminotransferase (Units/L)	64	15–37
Alanine aminotransferase (Units/L)	68	12–78
Total alkaline phosphatase (Units/L)	99	45–117
Total protein (g/dL)	7.5	6.4–8.2
Albumin (g/dL)	4.5	3.4–5.0
C-reactive protein (mg/dL)	1.84	0-0.300
Pro-B-natriuretic peptide (pg/mL)	187	Missing
White blood cell count (K/mm^3^)	8.3	4.1–9.3
Hemoglobin (gm/dL)	15.8	13.8–17.2
Hematocrit (%)	46.0	40.6–51.8
Platelet count (x 10^3^/µL)	290	150–450
Erythrocyte sedimentation rate (mm/hr)	11	0–15
COVID-19 (NAA)	Negative	Negative

**Figure 1:**
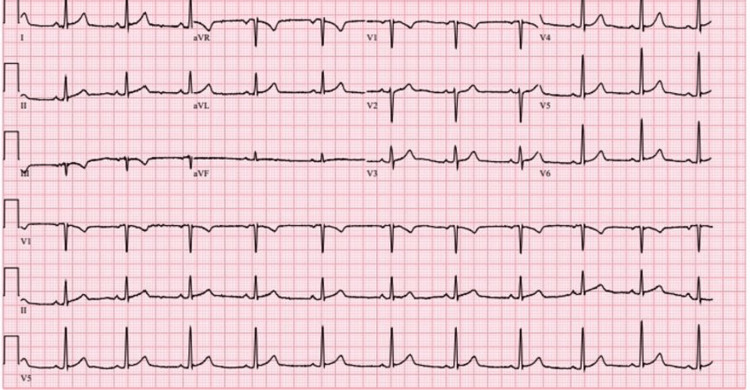
Patient's electrocardiogram

The chest radiograph was negative for any acute pathology. Chest computed tomography did not show any evidence of main or segmental pulmonary emboli. The echocardiogram did not reveal any evidence of vegetation. As the patient was getting ready to be transferred for admission to the main hospital, his urine drug screen results came back, which was positive for cocaine. The patient had previously specifically denied drug use but accepted it once the test results came back. His electrocardiogram showed sinus rhythm without any acute ST-T wave elevations (Figure 1).

**Figure 2:**
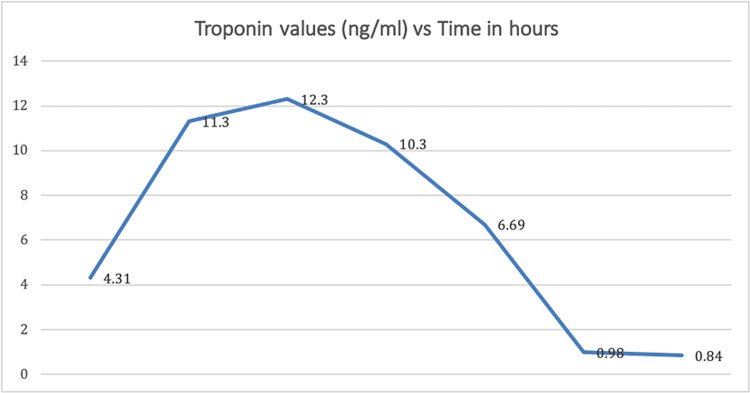
Patient's troponin levels over time

Troponin levels were high on the initial presentation, with a marked increase over subsequent draws. Over a treatment time of 62 hours, the troponin I levels reached as high as 12.30 ng/mL and decreased to 0.84 ng/mL at discharge (Figure 2). Serial electrocardiograms did not demonstrate any acute ST-T wave changes. A coronary angiogram did not reveal any cardiovascular pathology. The patient continued to make an uneventful recovery. He was discharged home with information on resources for substance abuse rehabilitation.

## Discussion

This case presents a man who was originally worked up on suspicion of endocarditis. Echocardiogram revealed a lack of vegetation on the heart valves, lowering endocarditis on the differential. The patient tested positive for cocaine use, which can impair myocardial contractility and precipitate NSTEMI. Cocaine is the second most commonly used illicit drug in the United States, trailing behind marijuana [8]. Cocaine causes increased availability of dopamine, serotonin, and norepinephrine in the synaptic clefts [14]. This leads to mood changes manifesting as euphoria or agitation and an increased cardiac demand secondary to elevated heart rate and blood pressure. There is also a vasoconstrictive effect in the coronary arteries, which can lead to decreased oxygen supply and subsequent angina or cardiac ischemia. Patients with other predisposing factors such as atherosclerotic disease of the coronary arteries may be at more risk of suffering chest pain after cocaine use.

In a cross-sectional study conducted with cocaine users, 32% of the participants reported having cocaine-related chest pains their entire life. About 78.5% of these subjects were men with an average age of 37.5 years, and 60.1% of them used cocaine every day. The study also reported an association between increased use of cocaine and increased risk of AMI [15]. The lingering effects of ischemia can last for a lifetime, if not treated correctly. 

A controversial treatment for cocaine-related chest pains is the use of beta-blockers. A 2018 meta-analysis was conducted on 1,794 different cocaine users, which showed that beta-blockers do not improve the symptoms of the patients in a significant way [16]. The evaluation of cocaine-induced chest pain in the ED should be similar to that of patients with suspected acute coronary syndrome (ACS). Due to the interrelationship between neuropsychiatric symptoms and cardiovascular complications, cocaine users should be given intravenous benzodiazepines [15].

## Conclusions

Cocaine use can cause myocardial infarction in young people. Following continued usage of cocaine, severe heart attacks, blood clots, and other cardiac ischemic injuries are liable to occur. Patients should be evaluated as if they were presenting with ACS, but they should also be given benzodiazepines to offset the neuropsychiatric symptoms that play a role in cardiac demand. Patients should be educated about the risks of cocaine abuse and provided with social tools that would facilitate the cessation of drug use.
